# What Has Become of

**Published:** 1917-12

**Authors:** 


**A Monthly Review of Medicine and Surgery**

EDITOR AND PUBLISHER, DR. A. L. BENEDICT, 228 Summer St., corner of Elmwood Ave., Buffalo, (Address for all communications. Please make personal and telephone calls before 1 P. M.)

**Third, in age, on the western continent. Independent, but supports professional organizations. News limited mainly to Western N. Y. and Penn.**

**Subscription $2.00 a year; $3.00 for two years in advance. Changes and errors in address or failure or duplication of delivery, should be reported immediately.**

Yearly Volume 73 DECEMBER, 1917 Number 5

### What Has Become Of

Cheap wood alcohol, that any one could distill from potato peelings, weeds and other vegetable substances containing cellulose, by a simple, cheap, portable apparatus?

Gasoline obtained by a method, approved by government experts, from less volatile parts of crude petroleum?

Gasoline derived from shale?

The green powder, pronounced practical by Henry Ford, to be added to water at a cost of a cent a gallon, to drive automobiles?

The method to make dyes from autumn leaves, also said to have been approved by the government?

The motor that would pick up aerial electric current and render power available to any one possessing the machine?

The numerous internal antiseptics, non-toxic and producing absolute sterilization?

The magnetic submarine destroyer exploding by pressure of water when the submarine descended below the lowest level of a floating vessel?

